# Suicide among Reproductive-Age Women in Northwest Ethiopia: A Community-Based Cross-Sectional Study

**DOI:** 10.1155/2024/1735716

**Published:** 2024-05-22

**Authors:** Techilo Tinsae, Biruk Fanta Alemayehu, Wondale Getinet Alemu

**Affiliations:** University of Gondar College of Medicine and Health Science, Department of Psychiatry, Gondar, Ethiopia

## Abstract

**Background:**

Suicide is one of the global burdens of morbidity and mortality in all reproductive-age women population groups across the world. It is one of the most significant contributors to the global burden of illness and a cause of morbidity. This study is aimed at finding out how it occurred and what risk factors were related to suicidal ideation and attempts among women in the reproductive-age group.

**Methods:**

A community-based cross-sectional study design was conducted using a multistage cluster sampling technique to get a total of 590 study participants from March to April 2021. Data were entered into EpiData version 3.1 and analyzed in bivariate and multivariable logistic regression models using Statistical Package for Social Science (SPSS) version 20. Variables with a *p* value <0.05 were declared to be associated risk factors with the outcome variable.

**Results:**

A total of 590 of the 598 sampled women participated, with a response rate of 98.7%. A one-month prevalence of suicidal ideation and attempt was 2.9% (95% CI: 1.5%, 4.4%) and 2.5% (95% CI: 1.4%, 3.7%), respectively. Intimate partner violence (AOR = 4.69, 95% CI: 1.53, 14.45), depression (AOR = 3.11, 95% CI: 1.11, 9.85), and history of mental illness (AOR = 5.18, 95% CI: 1.55, 17.32) were associated risk factors for suicide ideation. Anxiety (AOR = 3.55, 95% CI: 1.17, 10.81), being unmarried (AOR = 4.39, 95% CI: 1.49, 12.87), and history of mental illness (AOR = 7.95, 95% CI: 2.42, 26.15) were associated risk factors for suicide attempts.

**Conclusion:**

SI and SA are prevalent in reproductive-age women. Intimate partner violence, depression, anxiety, a history of mental illness, and being single were associated factors. This implies that providing relevant information, education, and continuing support is very crucial for reproductive-age group women to decrease the incidence and risk factors of suicide.

## 1. Introduction

The word suicide is derived from the Latin word suicidium, meaning “self-murder.” It is a fatal act that represents the person's wish to die [1, 2]. It is the deliberate infliction of death by hanging, poisoning, jumping, or injuring to kill oneself [2–4]. A suicide attempt (SA) is an act of nonfatal injury that is intentionally initiated and performed by the individual in the absence of outside assistance [2–4]. Suicidal ideations (SI), often called suicidal thoughts or ideas, are a broad term used to describe a range of contemplations, wishes, and preoccupations with death [3, 5].

Globally, the rates of attempted suicide are higher among women than in men [6]. According to a Chinese study, women were approximately 2.2 times more likely to attempt suicide than men throughout their lifetimes [7]. Among women, it is one of the most significant contributors to the global burden of illness and a cause of morbidity [8]. The prevalence of suicidal attempts and ideation among women in the community ranged from 0.8% to 29% [8]. Moreover, it can cause harmful effects such as injury, hospitalization, and loss of freedom, and it can put a billion-dollar financial burden on society [9, 10]. Numerous studies conducted in Ethiopia have shown the prevalence and risk factors for suicide behavior in various populations; however, research on women who are of reproductive age has received less attention. The distribution of health resources, the creation of relevant policies, the application of efficient treatment and preventive interventions, and the decrease in poor SA-related health outcomes among women of reproductive age all depend on a fuller understanding of SI and SA patterns.

Women who have SI and SA are subject to several negative outcomes. For example, suicide has been associated with 2.2-13% of maternal deaths [11–13], an increased risk of early labor and cesarean delivery, and an increased requirement for blood transfusions [14, 15]. Even though more than 75% of suicides occur in low- and middle-income countries, it is also the most serious problem in higher-income countries [9, 16].

During the postpartum period, the incidence of suicidal attempts is higher and ranges from 4% to 17.6% [17, 18]. SA and plans are much more prevalent in women with mental illnesses [19–21], and they are one of the main causes of death for women with psychopathological disorders [22, 23]. However, little is known about the prevalence or risk factors of SA and plans in women of reproductive age, in Ethiopia, in particular. Major risk factors for SI and SA included depression, exposure to potentially fatal situations, marital violence and mistreatment of children, low socioeconomic position, and a lack of social support [19, 20, 23, 24]. There is a 20% to 35% rise in suicide rates among people with major depressive disorder [25]. Numerous academics have recommended that social and health organizations try to identify women who are at high risk of suicide and implement significant initiatives to prevent suicide behavior [26]. Even though reproductive-age women are at higher risk for suicidal behavior, there is no community-based evidence regarding the problem. Therefore, this study was designed to estimate the prevalence and risk factors of suicidal ideation and attempts among reproductive-age women in northwest Ethiopia.

## 2. Methods and Materials

### 2.1. Study Design, Period, and Area

A community-based cross-sectional study design was employed from March to April 2021. The study was conducted in the central Gondar zone, northwest Ethiopia. The State of Amhara is located in the northwestern and north-central parts of Ethiopia. Bahir Dar is the capital city of the Amhara regional state, and it is located 560 kilometers from the capital city of Ethiopia. The Amhara regional state shares common borders with Oromiya in the south, Tigray in the north, Afar in the east, Benishangul/Gumuz in the southwest, and the Republic of Sudan in the west. According to the 1994 census, Amhara Regional's population was 13,834,297, of which 6,886751 were females and 6,947,546 were males [27].

### 2.2. Source and Study Population

All reproductive-age women (15–49 years) living in central Gondar and accessible throughout the data collection periods were the source population for this study, and reproductive-age women who were living 6 months and older in the central Gondar zone during the data collection period were the study population for this study [28].

### 2.3. Inclusion and Exclusion Criteria

All the women living in the reproductive-age group who were in the central Gondar zone were included in this study, and the reproductive-age group women who were critically sick during the data collection period and the women whose ages were less than 15 years and above 49 years were excluded from this study.

### 2.4. Sample Size Determination and Sampling Procedures

The sample size was determined using the following assumptions: *p* = 2.9% from the previous study [8], a 5% margin of error, and a 95% confidence interval. Therefore, *n* = (*Zα*/2)2∗*P* (1 − *P*)/*d*2 = (1.96)2∗(0.029)∗(0.971)/(0.02)2 = 271. A multistage cluster sampling approach was employed in the current study to recruit participants. The multistage sampling approach's potential for intercluster variability is taken into consideration by the design effect. The number of stages that were required to reach the final respondents in this case is intended to be comparable to the design effect. However, due to the study's low funding, we were only able to apply our design impact of 2. A 10% nonresponse rate was also taken into account, and 598 was the final sample size determined. During the process of attending to the participants, a lottery method was administered to reach out to the required three woredas (woreda is an administrative division or district in Ethiopia, which is further subdivided into kebeles (neighborhoods or communities), and 15 kebeles (kebele is a term used in Ethiopia for the smallest administrative unit or neighborhood within a woreda) (5 kebeles in each randomly selected woredas) were randomly selected (East Denbiya, Gondar Zuria, and Wogera). Finally, all women living between the ages of 15 and 49 were included in each cluster. The lottery method was used to select the first residence at random. If more than one woman of reproductive age was discovered in the chosen home, only one was invited to participate in an interview by lottery. If there are not any eligible women of reproductive age at the house we have picked, or if the house we have chosen is closed for another visit, we move on to the next house until we find an eligible candidate.

### 2.5. Data Collection Tools

#### 2.5.1. Suicidal Ideation and Attempt

The World Health Organization Composite International Diagnostic Interview (CIDI) [29] was used, which is a standard tool used to assess the prevalence and associated factors of suicide ideation and attempts. These behaviors were investigated using a hierarchy of single-item questions: lifetime suicidal ideation: “Have you ever thought of killing yourself?” If the answer is yes, the respondent is considered to be having suicidal thoughts. Current suicide ideation: “Have you seriously considered dying by suicide within the last month?” If the answer is yes, the respondent is considered to be having current suicidal ideation. Lifetime suicidal attempt: “Have you ever attempted suicide?” If the answer is yes, the respondent is considered to be making a lifetime suicidal attempt. Current suicidal attempt: “Have you attempted suicide within the last month?” If the answer was yes, the respondent was considered to be having a current suicidal attempt.

#### 2.5.2. Depression, Anxiety, and Stress Scale (DASS-21)

The reduced depression, anxiety, and stress (DASS-21) tools are used to assess depression, anxiety, and stress, which were developed by PF Lovibond and SH Lovibond [30]. DASS-21 has 21 items and three dimensions: depression (items 3, 5, 10, 13, 16, and 21), anxiety (items 2, 4, 7, 9, 15, and 20), and stress (items 1, 6, 8, 11, and 12). The scale for answers ranges from 0 (did not apply at all) to 3 on a four-point Likert-type scale (applied a lot or most of the time) [30, 31]. Reliable results have been obtained from numerous examinations of the psychometric properties of this scale [32–35]. Adult nonclinical and clinical samples have confirmed the validity and reliability of the DASS and DASS-21 [34]. The DASS-21 scale's total Cronbach's alpha was 0.74 [36] (Table 1). The women who scored moderate, severe, and extremely severe were used to categorize or level depression, anxiety, and stress based on the following cutoffs: the participants who scored low in the current study were considered to have no depression, anxiety, or stress. Individuals scoring less than 21 were classified as not depressed, while those scoring more than or equal to ≥21 were classified as depressed. Similarly, those scoring less than 15 were classified as not anxious, and those scoring more than 15 as anxious; those scoring less than 19 were classified as not stressed, and those scoring more than 19 as stressed [37].

#### 2.5.3. Intimate Partner Violence

The Women's Abuse Screening Test, developed by Coker et al. [38], was a useful tool for measuring intimate partner violence. It is the act of one or more physical violence (such as slapping, pushing, shoving, pulling, throwing something that could hurt, choking, burning on purpose, hitting the abdomen with a fist or with something else, and if a gun, knife, or any other weapon was used against a woman by an intimate partner) [39], emotional violence (such as insult, humiliation, intimidate, or scared on purpose by an intimate partner) [40], or sexual violence (such as experienced one or more acts or threats such as forced into sexual intercourse when she did not want, had sexual intercourse when she did not want to because she was afraid of what partner might do, and forced to do something sexual that she found degrading or humiliating by an intimate partner) [41]. Its rating levels range from 0 to 15, and a score of greater than 1 within a year indicates the presence of intimate violence [38]. The score ranges from 0 to 15, with a score of 1 indicating the presence of domestic violence [42].

#### 2.5.4. Perceived Social Support

The Oslo-3 social support scale was used to assess social support. Its scale has a total score of 14 and is divided into three areas. Those with a score of 3–8 were considered to have low social support, those with a score of 9–11 were considered to have moderate social support, and those with a score of 12–14 were considered to have favorable social support [43].

#### 2.5.5. Household Food Insecurity

Household food security was assessed using the Household Food Insecurity Scale (HFIAS). The frequency scores ranged from 0 to 3, while 0 was the score for nonoccurrence, 1 for rarely (once or twice in the past four weeks), 2 for sometimes (three to ten times in the past four weeks), and 3 for often (more than ten times in the past month) [44]. In this study, scores 0 were categorized as no and labeled as 0, and scores 1-9 were categorized as yes and labeled as 1 [45].

#### 2.5.6. Harmful Drinking

The Fast Alcohol Screening Test (FAST) scale, which was adapted from the Alcohol Use Disorders Identification Test (AUDIT) by Hodgson, contains four items with a rating score ranging from 0 to 16. A rating of >3 indicates dangerous drinking [46].

### 2.6. Data Collection Process

After reviewing relevant literature, the questionnaire was written in the English language and then translated into the Amharic language before being retranslated back into English to guarantee consistency. The study questionnaires were adapted from the World Mental Health Survey Initiative version of the World Health Organization Composite International Diagnostic Interview (CIDI) [29], which is a standard tool used to assess the prevalence and associated factors of suicide ideation and attempts. Before beginning the data collection procedure, fifteen health extension workers for data collection and three mental health professionals for supervisors were recruited, and five days of training were given to both data collectors and supervisors about the nature of the study objective, questionnaires, and how to interview study participants. Before, the actual data collection, the questionnaire was tested on 5% of the total sample size to check the difficulties encountered by the respondents as well as the data collectors, understandability, consistency, and time allocation. The internal consistency of the questionnaires was checked (Cronbach's alpha = 0.68). The data were checked for completeness, accuracy, and consistency by the investigators daily.

### 2.7. Data Analysis

The data was cleaned and cross-checked before analysis. The data was coded and entered into EPI Data Version 3.1 (which was needed to command values for variables and help to reduce data input mistakes compared to the statistical package for social sciences) before being exported to SPSS Version 20 for analysis. Frequency means and medians were calculated to summarize the sociodemographic and clinical characteristics of study participants and presented in the form of tables and texts. To satisfy the chi-square assumption, a crosstab was performed between the outcome variables and the independent variables, and a variable that satisfied the chi-square assumption was included in the logistic regression analysis. The binary logistic regression model was used to find factors linked to suicidal thoughts and attempts in women of reproductive age. All explanatory variables with a *p* value less than 0.2 in the bivariate logistic regression model were entered into the multivariate logistic regression model to control the effects of probable confounding factors. In the final model, the explanatory variables associated with suicidal ideation and attempts were identified using the AOR with a 95% CI and a *p* value less than or equal to 0.05. The tolerance is >0.1, and the variance inflated factor (VIF) is <10. The goodness of fit of the model was determined with the Hosmer and Lemeshow test, in which a *p* value greater than 0.05 was considered a statistical fit of the model. Suicide ideation had a model goodness score of 0.525, and the actual suicide attempt had a score of 0.929.

### 2.8. Ethical Consideration

Ethical clearance was obtained from the University of Gondar College of Medicine and Health Science Institutional Review Board (IBR) with a reference number of V/P/RCS/091/26/2028/13. A formal letter of administrative approval was obtained from each cluster health office. Written informed consent was obtained from each study participant, whose age was above eighteen, and a written informed assent from parents/guardians after a clear explanation of the purpose and aim of the study.

## 3. Results

### 3.1. Sociodemographic Factors

Among a total of 598 participants, 590 women fully responded to the given questionnaires with a 98.6% response rate. The remaining eight recruited participants did not complete all of the questionnaires and were therefore excluded from the analysis. The mean (±SD) age of respondents was 31.93 (±7.95) years. In this study, the majority of respondents (585 (99.2%)) were Orthodox, and 473 (80.3%) participants were currently married (Table 2).

### 3.2. Psychosocial and Alcohol-Related Factors

This study found that 44.2% of the respondents were exposed to stressful life events (Table 3).

### 3.3. Clinical-Related Factors

This study found that among reproductive-age women, 11.4% had depression, 5.8% had a history of mental illness, and 12.7% had anxiety (Table 4).

#### 3.3.1. Prevalence of Suicidal Ideation and Attempts in Reproductive-Age Women

The prevalence of one-month suicide ideation was 2.9% (95% CI: 1.5%, 4.4%). Among those who had a history of suicide ideation, 9 (1.5%) had a history of planning to die by suicide in their lifetime. The prevalence of one-month suicide attempts among reproductive-age women was 2.5% (95% CI: 1.4%, 3.7%). Of those who had one-month suicide attempts, 1.4% of participants had a history of trying to kill themselves one time, 0.8% of participants had a history of an attempt to die by suicide two times, and 0.3% of the respondents had a history of an attempt to kill themselves more than twice. The method that they used to die was suicide. 1.5% of the respondents used methods to kill themselves by hanging, 0.8% by taking poising, and 0.2% by using sharp materials. Concerning the seriousness of suicidal attempts, 1.4% of the participants reported having serious attempts, 0.8% tried to kill themselves, and 0.3% did not intend to kill themselves (Table 5).

#### 3.3.2. Determinants of Suicidal Ideation in Reproductive-Age Women

In multivariable logistic regression, intimate partner violence, mental illness history, depression, and being currently unmarried are variables linked with suicidal thoughts with a *p* value of 0.05.

The odds of suicidal ideation were 4.7 times more common among reproductive-age women who had experienced intimate partner violence (AOR = 4.69, 95% CI: 1.53, 14.45) either physically, psychologically, or sexually compared to those who had not experienced intimate partner violence. Women with depression were three times more likely to have suicidal ideation than those without depression (AOR = 3.11, 95% CI: 1.11, 9.85). Women with a history of mental illness were 5.2 times more likely to have suicidal ideation than women who had no history of mental illness (AOR = 5.18, 95% CI: 1.55, 17.32). In addition, the risk of suicidal ideation was 3.4 times more prevalent in single women than in married women (AOR = 3.37, 95% CI: 1.13, 10.03) (Table 6).

#### 3.3.3. Determinants of Suicidal Attempts among Reproductive-Age Women

In a multivariable logistic regression analysis, anxiety, mental illness history, and marital status were variables associated with suicidal attempts.

The odds of suicidal attempts were 3.6 times higher among women who had anxiety than among women who had no anxiety (AOR = 3.55, 95% CI: 1.17, 10.81). The odds of suicidal attempts were 4.4 times more likely among currently unmarried women (single, divorced, or widowed) than among married women (AOR = 4.39, 95% CI: 1.49, 12.87). The odds of suicidal attempts were 8 times higher among women who had a history of mental illness than among women who had no history of mental illness (AOR = 7.95, 95% CI: 2.42, 26.15) (Table 7).

## 4. Discussion

### 4.1. Suicidal Ideation

The results of this study offer important and previously unavailable details regarding the one-month prevalence and risk factors of suicidal ideation and attempts in reproductive-age women. The one-month prevalence of suicidal ideation among reproductive-age women was 2.9%. The result of this finding is consistent with previous similar studies conducted in Tanzania (4.3%, 4.2%) [8], the US (2.7%) [47], Brazil (4.3%) [8], Serbia (1.9%) [8], and Japan (2.1%) [8]. This indicates that suicidal ideation is more prevalent across the country in reproductive-age women. However, the magnitude of this study is lower than studies found in Peru (13.6% and 6.8%) [8], Namibia (8.9%) [8], Brazil (6.3%) [48], Thailand (9.9%, 6%) [8], South Africa (which reported 12%) [49], and Ethiopia (8.2%) [50, 51]. This discrepancy might be due to sociocultural differences, and sample population differences, or it may be due to women's willingness to disclose suicidal ideation, which is culturally considered socially unacceptable.

According to the findings of this study, intimate partner violence was an associated risk factor for suicidal ideation. The results are supported by studies conducted in Tanzania [8], Peru [52], the USA [53], and India [54]. The possible reason for this association is the fact that intimate partner violence can cause a variety of consequences, including social discrimination and stigmatization, physical disability, school and job absenteeism, the failing economic dependency of the women, who may develop acute stress disorders, and posttraumatic stress disorder, as well as depression. In this study, depression is another associated risk factor for suicidal ideation. The result is supported by studies done in Brazil [55], Pakistan [56], America [57], China [58], Egypt [59], the USA [47], and South Africa [60]. This might be due to depression, which can cause loss of self-esteem, sadness, hopelessness, decreased happiness in one's life, poor self-control, a feeling of helplessness, and negative expectations of oneself and the future. This paper found that women who had a history of mental illness had higher odds of suicidal ideation than those who had no such history. The results were supported by studies done in southern Ethiopia [50] and New York [61]. The possible reasons for this association should be that mental health problems may have a variety of impacts on interactive as well as multiplicative effects, such as leading to stigma and discrimination, command hallucinations or delusions that can cause thoughts of suicidal ideation, the expectation of the chronic nature of the mental disorder, the social and cultural view of mental illness and loss of marriage, economic and social status, occupation, and school due to this problem. This study discovered that unmarried women were more likely to have suicidal thoughts than married women. This study is supported by a study done in Brazil [62], the Caribbean [63], and Asia [64]. The possible reason is that being divorced or widowed may trigger feelings of loneliness, inferiority, negligence, a sense of failure, depression, loss of emotional and psychological support, and an increased economic burden caused by being single, divorced, or widowed.

### 4.2. Suicidal Attempts

This study revealed that the magnitude of the one-month prevalence of suicidal attempts in reproductive-age women was 2.5%. The magnitude of the one-month prevalence of suicide attempts is consistent with findings reported from Asian Americans at 2.5% [65], and southern Ethiopia at 2.7% [50]. However, the result of this study is lower than previous studies reports from South Africa at 6% [49] and America at 6% [57]. The possible explanation for this discrepancy might be due to sampling population differences, sociocultural factors, level of mental health awareness, attitudes towards mental illness, and study settings. Regarding risk factors, women who had anxiety symptoms were more likely to attempt suicide than those who had no anxiety symptoms. This result was supported by studies done in Pakistan [56], America [57, 65], and Egypt [59]. The possible justification for this associated risk factor, anxiety, should cause apprehensive expectations, difficulty concentrating, stress, and difficulty sleeping, and they may consider suicidal attempts as a peaceful escape from an apprehensive situation. A history of mental illness was another risk factor associated with suicidal attempts in reproductive-age women. The result is consistent with studies conducted in America [61], the US [66], and the World Mental Health Survey [67]. The possible explanations might be that mental illness can cause social discrimination, impairment of job performance due to mental illness, divorce from a spouse, the feeling of loneliness, negative expectations for the future, and the economic burden of treatment, all of which may cause suicidal attempts. Being single, divorced, or widowed was positively associated with suicidal attempts and supported by studies done in the US [65]. The possible reason for this may be that being single or divorced leads to emotional instability, a feeling of helplessness and hopelessness, and an increased economic burden because most women depend on their husband's income.

### 4.3. Limitation

Because of the cross-sectional study design, it cannot establish a temporal relationship between outcome variables and significant associated factors. The second limitation of this study was that it was not generalized to all age groups of women. The lack of comparison groups was another limitation of this study. In addition, the sensitive nature of disclosing the problem may produce a social desirability bias, which could underestimate the magnitude of the problem. So, the authors recommended for future researchers to do advanced longitudinal studies.

## 5. Conclusion

This study found that suicidal ideation and suicide attempts were high when compared to previous studies. Intimate partner violence, depression, a history of mental illness, anxiety, and currently being single were all strongly linked to suicidal ideation and attempts. This indicates that providing relevant information, education, and continuous support to women is very crucial. For future researchers, we recommend an advanced study design that would strongly infer the causal link between exposure to varying forms of independent factors and the development of suicidal behaviors. In addition, we recommend qualitative research to explore the feelings after a suicidal attempt in depth.

## Figures and Tables

**Table 1:** Depression, anxiety, and stress scales (DASS-21).

Ratings	Depression	Anxiety	Stress
Normal	0-9	0-7	0-14
Mild	10-13	8-9	15-18
Moderate	14-20	10-14	19-25
Severe	21-27	15-19	26-33
Extremely severe	28+	20+	34+

**Table 2:** Distribution of sociodemographic factors in reproductive-age women in Northwest Ethiopia, 2021 (*n* = 590).

Variables	Frequency	Percent
Age		
15-19	38	6.4
20-35	359	60.4
36 and above	193	32.7
Age in the first marriage		
<18 years	265	44.9
18 and above	249	42.2
Marital status		
Currently married	473	80.3
Currently unmarried	117	19.8
Educational status		
No formal education	280	47.5
Primary education	139	23.6
High school and above	171	29.0
Living status		
Living with a husband	433	73.4
Living with other relatives	157	26.6
Residence		
Rural	475	80.5
Urban	115	19.5
Religion		
Orthodox	585	99.2
Muslim	3	0.5
Catholic	2	0.3
Ethnicity		
Amhara	572	69.9
Tigre	1	0.2
Qemant	17	2.9
Number of children		
No children	104	17.6
Have children	486	82.4
Current pregnancy		
Yes	90	84.7
No	500	15.3

**Table 3:** Distributions of psychosocial factors in reproductive-age women in Northwest Ethiopia, 2021 (*n* = 590).

Variable	Frequency	Percent
Exposure to stressful life events		
Yes	261	44.2
No	329	55.8
Intimate partner violence		
Yes	209	35.4
No	381	64.6
Social support		
Poor	85	14.4
Moderate	277	46.9
Strong	228	38.6
Hazardous alcohol use		
Yes	92	15.6
No	498	84.4

**Table 4:** Distributions of clinical and substance-related factors of reproductive-age women in Northwest Ethiopia, 2021 (*n* = 590).

Variables	Frequency	Percent
Depression		
Yes	67	11.4
No	523	88.6
Anxiety		
Yes	75	12.7
No	515	87.3
Stress		
Yes	9	1.5
No	581	98.5
History of mental illness		
Yes	34	5.8
No	556	94.2
Gynecological problem		
Yes	29	4.9
No	561	95.1
History of abortion		
Yes	96	16.3
No	494	83.7

**Table 5:** Frequency distribution of suicide ideation and attempt in reproductive-age women in Northwest Ethiopia, 2021 (*n* = 590).

Variables	Frequency	Percent
Suicidal ideation in the last month		
Yes	17	2.9
No	573	97.1
Last month's suicide plan		
Yes	9	1.5
No	581	98.5
Suicidal attempts in the last month		
Yes	15	2.5
No	575	97.5
One-month frequency of suicide attempts		
Once	8	1.4
Twice	5	0.8
More than twice	2	0.3
Methods used for suicide attempts		
Hanging	9	1.5
Poisoning	5	0.8
Using sharp material	1	0.2
Which describes your suicide?		
A serious attempt	8	1.4
Tried to kill me	5	0.8
I did not intend to die	2	0.3

**Table 6:** Multivariate logistic regression analysis on suicide ideation in reproductive-age women in Northwest Ethiopia, 2021 (*n* = 590).

Variables	Suicidal ideation		COR (95% CI)	AOR (95% CI)	*p* value
	Yes	No			
Intimate partner violence					
Yes	12	196	4.62 (1.60, 13.29)	4.69 (1.52, 14.45)	0.007
No	5	377	1		
Marital status					
Currently married	9	464	1	3.37 (1.13, 10.03)	0.029
Currently unmarried⁣^∗^	8	109	3.78 (1.43, 10.03)		
Anxiety					
Yes	8	67	6.71 (2.51, 17.99)	2.64 (0.76, 9.19)	0.129
No	9	506	1		
Depression					
Yes	8	59	7.74 (2.88, 20.84)	3.31 (1.11, 9.85)	0.031
No	9	514	1		
Hazardous alcohol abuse					
Yes	5	87	2.33 (0.80, 6.77)	1.04 (0.30, 3.59)	0.956
No	12	486	1		
History of mental illness					
Yes	5	29	7.82 (2.58, 23.67)	5.18 (1.55, 17.32)	0.008
No	12	544	1		
Living status					
Living with a husband	9	148	1	1.76 (0.33, 9.42)	0.508
Living with other relatives	8	425	3.23 (1.22, 8.53)		
Participant's occupation being housewife	9	210	1		
Not being a housewife⁣^∗∗^	8	363	1.94 (0.74, 5.12)	1.08 (0.37, 3.13)	0.893

⁣^∗^Single, divorced, and widowed; ⁣^∗∗^government worker, private worker, merchant, student, and no job.

**Table 7:** Multivariate logistic regression analysis on suicide attempts among reproductive-age women in Northwest Ethiopia, 2021 (*n* = 590).

Variables	Suicidal attempt		COR (95% CI)	AOR (95% CI)	*p* value
	Yes	No			
Anxiety					
Yes	6	69	4.89 (1.69, 14.16)	3.55 (1.17, 10.81)	0.026
No	9	506	1		
Living status					
With husband	9	148	1	3.03 (0.92, 10.03)	0.069
With other relatives	6	427	4.33 (1.52, 12.38)		
Marital status					
Currently married	7	466	1	4.39 (1.49, 12.87)	0.007
Currently unmarried⁣^∗^	8	109	4.89 (1.74, 13.76)		
History of mental illness					
Yes	5	29	9.41 (3.02, 29.34)	7.947 (2.42, 26.15)	0.001
No	10	549	1		
Intimate partner violence					
Yes	9	199	2.83 (0.99, 8.08)	0.03 (0.92, 10.03)	0.069
No	6	376	1		
Depression					
Yes	7	60	7.51 (2.63, 21.44)	2.17 (0.57, 8.26)	0.256
No	8	515	1		
Occupation					
Housewife	9	210	1		
Others	6	365	2.61 (0.92, 7.43)	1.43 (0.45, 4.49)	0.546
Oral contraceptive					
Yes	5	301	0.46 (0.15, 1.35)	1.02 (0.15, 4.19)	0.981
No	10	274	1		

⁣^∗^Single, divorced, and widowed.

## Data Availability

The main parts of the data generated during this study are included in this article. Other data will be available from the corresponding author upon request.

## Abbreviations

AAS: Abuse assessment screen
ACAS: Audio computer-assisted self-interview
AOR: Adjusted odds ratio
AUDIT: Alcohol use disorder investigation test
CIDI: Composite international diagnostic interview
EPDS: Edinburgh Postnatal Depression Scale
ETB: Ethiopian birr
DASS-21: Depression, Anxiety, and Stress Scale-21 Items FAST stands for Fast Alcohol Screening Tool
HFIAS: Household Food Insecurity Access Scale
HIV: Human immunodeficiency virus
MINI: Mini International Neuropsychiatric Interview
OSS: Oslo Social Support Scale
US: United States
WHO: World Health Organization
WASH: Women's Abuse Screening Test
WMH-CIDI: World Mental Health Composite International Diagnostic Interview
WMH: World mental health.

## References

[1] American Psychiatric Association D (2013). *Diagnostic and statistical manual of mental disorders: DSM-5*.

[2] Jans T., Taneli Y., Warnke A. (2012). *Suicide and self-harming behavior*.

[3] Association AP (2013). *Diagnostic and Statistical Manual of Mental Disorders*.

[4] O’Carroll P. W., Berman A. L., Maris R., Moscicki E., Tanney B., Silverman M. (2002). Beyond the Tower of Babel. *Suicide Prevention: The Global Context*.

[5] Goodfellow B., Kolves K., De Leo D. (2018). Contemporary nomenclatures of suicidal behaviors: a systematic literature review. *Suicide and Life‐Threatening Behavior*.

[6] Vijayakumar L. (2017). Challenges and opportunities in suicide prevention in South-East Asia. *WHO South-East Asia Journal of Public Health*.

[7] Cao X.-L., Zhong B.-L., Xiang Y.-T. (2015). Prevalence of suicidal ideation and suicide attempts in the general population of China: a meta-analysis. *The International Journal of Psychiatry in Medicine*.

[8] Devries K., Watts C., Yoshihama M. (2011). Violence against women is strongly associated with suicide attempts: evidence from the WHO multi-country study on women’s health and domestic violence against women. *Social Science & Medicine*.

[9] Klonsky E. D., May A. M., Saffer B. Y. (2016). Suicide, suicide attempts, and suicidal ideation. *Annual Review of Clinical Psychology*.

[10] Takahashi Y., Takahashi S., Imamura Y., Yamashita R. (2014). The national strategies for suicide prevention by the United Nation/World Health Organization and the present situation of suicide in the East Asia. *Seishin Shinkeigaku Zasshi=Psychiatria et Neurologia Japonica*.

[11] Fildes J., Reed L., Jones N., Martin M., Barrett J. (1992). Trauma. *Journal of Trauma and Acute Care Surgery*.

[12] Dannenberg A. L., Carter D. M., Lawson H. W., Ashton D. M., Dorfman S. F., Graham E. H. (1995). Homicide and other injuries as causes of maternal death in New York City, 1987 through 1991. *American Journal of Obstetrics and Gynecology*.

[13] Lindahl V., Pearson J. L., Colpe L. (2005). Prevalence of suicidality during pregnancy and the postpartum. *Archives of Women’s Mental Health*.

[14] Gentile S. (2011). Suicidal mothers. *Journal of Injury and Violence Research*.

[15] Högberg U., Innala E., Sandström A. (1994). Maternal mortality in Sweden, 1980-1988. *Obstetrics & Gynecology*.

[16] Arensman E., Scott V., De Leo D., Pirkis J. (2020). Suicide and Suicide Prevention from a Global Perspective. *Crisis*.

[17] Howard L. M., Flach C., Mehay A., Sharp D., Tylee A. (2011). The prevalence of suicidal ideation identified by the Edinburgh Postnatal Depression Scale in postpartum women in primary care: findings from the RESPOND trial. *BMC Pregnancy and Childbirth*.

[18] Burgut F. T., Bener A., Ghuloum S., Sheikh J. (2013). A study of postpartum depression and maternal risk factors in Qatar. *Journal of Psychosomatic Obstetrics & Gynecology*.

[19] Johannsen B. M. W., Larsen J. T., Laursen T. M., Bergink V., Meltzer-Brody S., Munk-Olsen T. (2016). All-cause mortality in women with severe postpartum psychiatric disorders. *American Journal of Psychiatry*.

[20] Castro e Couto T., Brancaglion M. Y., Cardoso M. N. (2016). Suicidality among pregnant women in Brazil: prevalence and risk factors. *Archives of Women's Mental Health*.

[21] Tinsae T., Shumet S., Azale T. (2024). Exposure to stress-full life events and help-seeking behaviors among reproductive-age women in Northwest Ethiopia: community-based cross-sectional study. *Journal of Affective Disorders*.

[22] Madhavan I., Kareparambil Balan R., Neeratty Asokan B., Mekkattukunnel Andrews A., Valliot A. (2019). A retrospective analysis of pattern of suicide in autopsied cases in a tertiary care hospital. *Asia Pacific Journal of Medical Toxicology*.

[23] Gressier F., Guillard V., Cazas O., Falissard B., Glangeaud-Freudenthal N. M., Sutter-Dallay A.-L. (2017). Risk factors for suicide attempt in pregnancy and the post-partum period in women with serious mental illnesses. *Journal of Psychiatric Research*.

[24] Tavares D., Quevedo L., Jansen K., Souza L., Pinheiro R., Silva R. (2012). Prevalence of suicide risk and comorbidities in postpartum women in Pelotas. *Revista Brasileira de Psiquiatria*.

[25] Angst J., Angst F., Stassen H. H. (1999). Suicide risk in patients with major depressive disorder. *Journal of Clinical Psychiatry*.

[26] Belete H., Misgan E. (2019). Suicidal behaviour in postnatal mothers in northwestern Ethiopia: a cross-sectional study. *BMJ Open*.

[27] Hudson G. (1999). Linguistic analysis of the 1994 Ethiopian census. *Northeast African Studies*.

[28] Yasmin S., Mukherjee A. (2012). A cyto-epidemiological study on married women in reproductive age group (15-49 years) regarding reproductive tract infection in a rural community of West Bengal. *Indian Journal of Public Health*.

[29] Kessler R. C., Üstün T. B. (2004). The world mental health (WMH) survey initiative version of the World Health Organization (WHO) composite international diagnostic interview (CIDI). *International Journal of Methods in Psychiatric Research*.

[30] Lovibond P. F., Lovibond S. H. (1995). The structure of negative emotional states: comparison of the depression anxiety stress scales (DASS) with the Beck depression and anxiety inventories. *Behaviour Research and Therapy*.

[31] Coker A., Coker O., Sanni D. (2018). Psychometric properties of the 21-item depression anxiety stress scale (DASS-21). *African Research Review*.

[32] Chan R. C., Xu T., Huang J. (2012). Extending the utility of the depression anxiety stress scale by examining its psychometric properties in Chinese settings. *Psychiatry Research*.

[33] Musa R., Fadzil M. A., Zain Z. (2007). Translation, validation and psychometric properties of Bahasa Malaysia version of the depression anxiety and stress scales (DASS). *ASEAN Journal of Psychiatry*.

[34] Antony M. M., Bieling P. J., Cox B. J., Enns M. W., Swinson R. P. (1998). Psychometric properties of the 42-item and 21-item versions of the depression anxiety stress scales in clinical groups and a community sample. *Psychological Assessment*.

[35] Brown T. A., Chorpita B. F., Korotitsch W., Barlow D. H. (1997). Psychometric properties of the depression anxiety stress scales (DASS) in clinical samples. *Behaviour Research and Therapy*.

[36] Moya E., Larson L. M., Stewart R. C., Fisher J., Mwangi M. N., Phiri K. S. (2022). Reliability and validity of depression anxiety stress scale (DASS)-21 in screening for common mental disorders among postpartum women in Malawi. *BMC Psychiatry*.

[37] Lovibond S. H., Lovibond P. F. (2013). *Depression Anxiety and Stress Scales (DASS)*.

[38] Coker A. L., Pope B. O., Smith P. H., Sanderson M., Hussey J. R. (2001). *Assessment of clinical partner violence screening tools*.

[39] Fekadu E., Yigzaw G., Gelaye K. A. (2018). Prevalence of domestic violence and associated factors among pregnant women attending antenatal care service at University of Gondar Referral Hospital, Northwest Ethiopia. *BMC Women's Health*.

[40] Liyew A. M., Alem A. Z., Ayalew H. G. (2022). Magnitude and factors associated with intimate partner violence against pregnant women in Ethiopia: a multilevel analysis of 2016 Ethiopian demographic and health survey. *BMC Public Health*.

[41] Bifftu B. B., Dachew B. A., Tadesse Tiruneh B., Zewoldie A. Z. (2017). Domestic violence among pregnant mothers in Northwest Ethiopia: prevalence and associated factors. *Advances in Public Health*.

[42] Rabin R. F., Jennings J. M., Campbell J. C., Bair-Merritt M. H. (2009). Intimate partner violence screening Tools. *American Journal of Preventive Medicine*.

[43] Dalgard O. S., Dowrick C., Lehtinen V. (2006). Negative life events, social support and gender difference in depression: a multinational community survey with data from the ODIN study. *Social Psychiatry and Psychiatric Epidemiology*.

[44] Hussein F. M., Ahmed A. Y., Muhammed O. S. (2018). Household food insecurity access scale and dietary diversity score as a proxy indicator of nutritional status among people living with HIV/AIDS, Bahir Dar, Ethiopia, 2017. *PloS One*.

[45] Shayo F. K., Lawala P. S. (2019). Does food insecurity link to suicidal behaviors among in-school adolescents? Findings from the low-income country of sub-Saharan Africa. *BMC Psychiatry*.

[46] Hodgson R., Alwyn T., John B., Thom B., Smith A. (2002). The FAST alcohol screening test. *Alcohol and Alcoholism*.

[47] Gavin A. R., Tabb K. M., Melville J. L., Guo Y., Katon W. (2011). Prevalence and correlates of suicidal ideation during pregnancy. *Archives of Women's Mental Health*.

[48] Huang H., Faisal-Cury A., Chan Y.-F., Tabb K., Katon W., Menezes P. R. (2012). Suicidal ideation during pregnancy: prevalence and associated factors among low-income women in São Paulo, Brazil. *Archives of Women's Mental Health*.

[49] Onah M. N., Field S., Bantjes J., Honikman S. (2017). Perinatal suicidal ideation and behaviour: psychiatry and adversity. *Archives of Women's Mental Health*.

[50] Belete K., Kassew T., Demilew D., Zeleke T. A. (2021). Prevalence and correlates of suicide ideation and attempt among pregnant women attending antenatal care services at public hospitals in southern Ethiopia. *Neuropsychiatric Disease and Treatment*.

[51] Zewdu L. B., Reta M. M., Yigzaw N., Tamirat K. S. (2021). Prevalence of suicidal ideation and associated factors among HIV positive perinatal women on follow-up at Gondar town health institutions, Northwest Ethiopia: a cross-sectional study. *BMC Pregnancy and Childbirth*.

[52] Ellsberg M., Jansen H. A., Heise L., Watts C. H., Garcia-Moreno C. (2008). Intimate partner violence and women's physical and mental health in the WHO multi-country study on women's health and domestic violence: an observational study. *The Lancet*.

[53] Alhusen J. L., Frohman N., Purcell G. (2015). Intimate partner violence and suicidal ideation in pregnant women. *Archives of Women's Mental Health*.

[54] Maselko J., Patel V. (2008). Why women attempt suicide: the role of mental illness and social disadvantage in a community cohort study in India. *Journal of Epidemiology & Community Health*.

[55] Farias D. R., Pinto T. . J. P., Teofilo M. M. A. (2013). Prevalence of psychiatric disorders in the first trimester of pregnancy and factors associated with current suicide risk. *Psychiatry Research*.

[56] Asad N., Karmaliani R., Sullaiman N. (2010). Prevalence of suicidal thoughts and attempts among pregnant Pakistani women. *Acta Obstetricia et Gynecologica Scandinavica*.

[57] Pilver C. E., Libby D. J., Hoff R. A. (2013). Premenstrual dysphoric disorder as a correlate of suicidal ideation, plans, and attempts among a nationally representative sample. *Social Psychiatry and Psychiatric Epidemiology*.

[58] Lee S., Fung S., Tsang A. (2007). Lifetime prevalence of suicide ideation, plan, and attempt in metropolitan China. *Acta Psychiatrica Scandinavica*.

[59] Moustafa M. A., Youssef U. M., Sleem N. F., Mohamed el- Hanafy R. (2019). Prevalence and associated factors of suicide among pregnant women at Zagazig university hospitals. *Zagazig University Medical Journal*.

[60] Rochat T. J., Bland R. M., Tomlinson M., Stein A. (2013). Suicide ideation, depression and HIV among pregnant women in rural South Africa. *Health*.

[61] Cooperman N. A., Simoni J. M. (2005). Suicidal ideation and attempted suicide among women living with HIV/AIDS. *Journal of Behavioral Medicine*.

[62] Botega N. J., Barros M. B. A., Oliveira H. B., Dalgalarrondo P., Marín-León L. (2005). Suicidal behavior in the community: prevalence and factors associated with suicidal ideation. *Brazilian Journal of Psychiatry*.

[63] Thanh H. T. T., Tran T. N., Jiang G.-X., Leenaars A., Wasserman D. (2006). Life time suicidal thoughts in an urban community in Hanoi, Vietnam. *BMC Public Health*.

[64] Vijayakumar L. (2015). Suicide in women. *Indian Journal of Psychiatry*.

[65] Cheng J. K. Y., Fancher T. L., Ratanasen M. (2010). Lifetime suicidal ideation and suicide attempts in Asian Americans. *Asian American Journal of Psychology*.

[66] Desai R. A., Liu-Mares W., Dausey D. J., Rosenheck R. A. (2003). Suicidal ideation and suicide attempts in a sample of homeless people with mental illness. *The Journal of Nervous and Mental Disease*.

[67] Nock M. K., Borges G., Bromet E. J. (2008). Cross-national prevalence and risk factors for suicidal ideation, plans, and attempts. *The British journal of Psychiatry*.

